# Transformation of patchouli alcohol to β-patchoulene by gastric juice: β-patchoulene is more effective in preventing ethanol-induced gastric injury

**DOI:** 10.1038/s41598-017-05996-5

**Published:** 2017-07-17

**Authors:** Yuhong Liu, Jiali Liang, Jiazhen Wu, Hanbin Chen, Zhenbiao Zhang, Hongmei Yang, Liping Chen, Haiming Chen, Ziren Su, Yucui Li

**Affiliations:** 1 Guangdong Provincial Key Laboratory of New Drug Development and Research of Chinese Medicine, Mathematical Engineering Academy of Chinese Medicine, Guangzhou University of Chinese Medicine, Guangzhou, 510006 China; 2 The First Affiliated Hospital of Chinese Medicine, Guangzhou University of Chinese Medicine, Guangzhou, 510405 China; 3 Guangdong Provincial Hospital of Chinese Medicine, Guangzhou University of Chinese Medicine, Guangzhou, 510120 China; 4 Dongguan Mathematical Engineering Academy of Chinese Medicine, Guangzhou University of Chinese Medicine, Dongguan, 523808 China; 5 College of Pharmacy, Jinan University, Guangzhou, 510632 China

## Abstract

Pogostemonis Herba is a functional food approved in Asian countries. Its major constituent, patchouli alcohol (PA), possesses a gastroprotective effect and is reported to transform into β-patchoulene (β-PAE) under acidic conditions. To investigate whether β-PAE, the metabolite of PA, has a protective effect on the gastrointestinal tract, the formation of β-PAE by gastric juice and the anti-ulcerogenic potential of β-PAE against ethanol-induced gastric injury were evaluated. The Results indicated that PA was converted to β-PAE by rat gastric juice. Additionally, β-PAE was significantly better than PA at reducing the area of gastric ulcer. The overproduction of malondialdehyde, tumour necrosis factor-α, interleukin (IL)-1β, IL-6, Fas, FasL and caspase-3 was markedly inhibited by β-PAE while the underproduction of superoxide dismutase, glutathione and catalase was significantly improved. β-PAE also regulated the NF-κB and ERK1/2 signalling pathways. Our findings suggest that β-PAE has potential therapeutic efficacy for antiulcer treatment.

## Introduction

Gastrointestinal health is a primary indicator of overall health. Ten percent of the world population suffers from gastric ulcer, 1% of which can deteriorate into cancer. Gastric ulcer is therefore extremely harmful to human health and is classified as a precancerous disease by the World Health Organization (WHO). This disease, characterized by necrosis, reduced blood flow, neutrophils infiltration, induction of oxidative stress and inflammatory mediator secretion, is predominantly caused by *Helicobacter pylori*, use of non-steroidal anti-inflammatory drugs (NSAIDs), alcohol, smoking and stress^1^. Alcohol consumption has been related to gastric mucosal lesions, including gastritis, gastric ulcer and even gastric carcinoma^2^. The underlying mechanisms of ethanol-induced gastric injury have not been well defined. However, growing evidence has demonstrated that oxidative stress, inflammatory cytokines and apoptosis play crucial roles in the pathogenesis of ethanol-induced gastric ulcer^3, 4^. Many synthetic drugs are available to treat gastric ulcer, but the development of sustainable natural agents exhibiting notable efficacy in preventing various ulcerative conditions is of great importance. There is growing interest in identifying and developing botanical extracts and phytochemicals from edible plants^5^.

**Figure 1:**
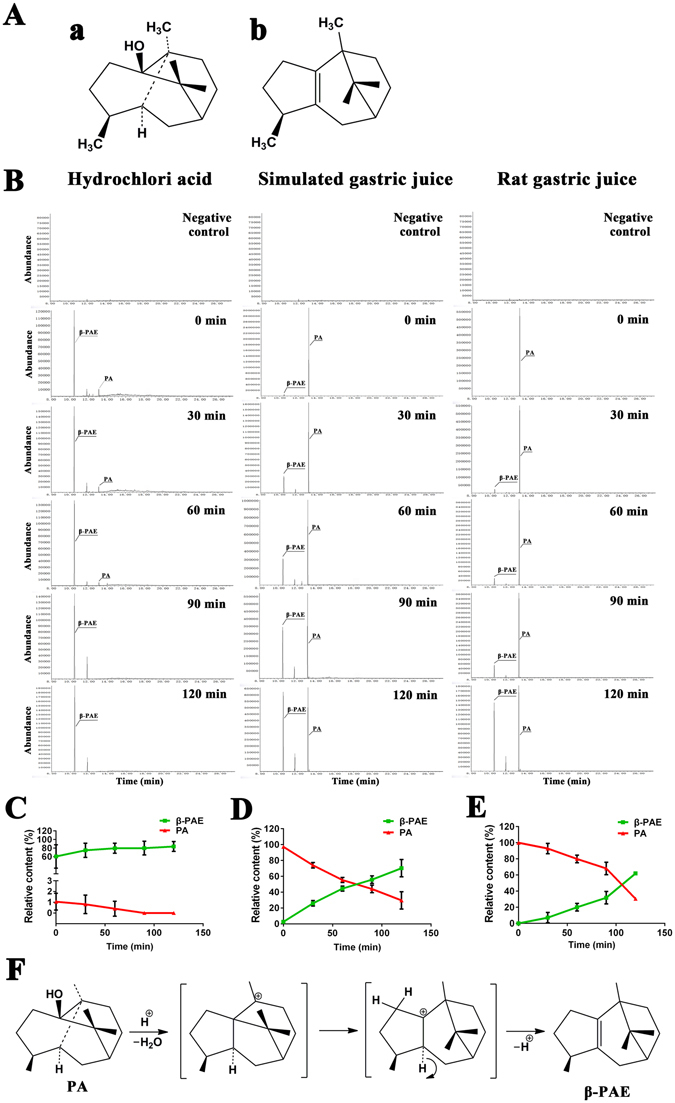
Transformation of PA into β-PAE by hydrochloric acid, stimulated gastric juice or rat gastric juice. (**A**) The structure of patchouli alcohol (a) and β-patchoulene (b). (**B**) Total ion chromatogram (TIC) of PA and β-PAE after transformation of PA in hydrochloric acid, stimulated gastric juice or rat gastric juice for 0, 30, 60, 90 and 120 min. Time course of transformation of PA into β-PAE by hydrochloric acid (**C**), simulated gastric juice (**D**) or rat gastric juice (**E**). Each point represents the means ± SD of three experiments. (**F**) Mechanism of transformation of PA into β-PAE.

*Pogostemon cablin* (Blanco) Benth. (Labiatae), commonly referred to as Cablin Patchouli or “Guang-Huo-Xiang”, is a well-known traditional healthy food and medicinal herb used in Asian countries. Its fresh leaves and dried powder are widely consumed in China and other Asian countries in the form of food flavour supplements, tea, beverages, candy, baked products, and common botanical ingredients in functional foods and dietary supplements^6, 7^. Substantial pharmacological studies have suggested anti-influenza virus, anti-inflammatory, analgesic, anti-emetic, antimicrobial and radical-scavenging activities for Pogostemonis Herba. Traditionally, Pogostemonis Herba has been widely applied for gastrointestinal disease in China as an important component of many popular Chinese herbal formulae, such as *Huoxiang Zhengqi Liquid* and *Baoji Pill* ^8^. As the principal constituent of Pogostemonis Herba, patchouli alcohol (PA, MW: 222.37, Fig. 1Aa) is considered to have bright prospects in the development of functional foods.

PA, a natural tricyclic sesquiterpene, has been shown to possess gastroprotective effect against gastric injury in rats induced by ethanol, indomethacin, water immersion and restraint stress in our previous study^9^. Nevertheless, the mechanism of action of PA has not been elucidated. It is well known that oral administration is the most common method of administration, and PA is often administered orally. Modern research has demonstrated that PA dehydrates and primarily rearranges to β-patchoulene (β-PAE, MW: 204.35, Fig. 1Ab) under acidic condition^10^. Thus, after oral administration, transformation of PA likely occurs in the stomach in the presence of acidic gastric juice. β-PAE is also an effective component of Pogostemonis Herba. The aforementioned findings prompted us to determine whether PA transforms to β-PAE in gastric juice and exerts a gastroprotective effect and whether β-PAE is more effective than PA in counteracting gastric ulcer. The metabolites of PA formed in gastric juice were analysed; and the possible anti-ulcerogenic effect of β-PAE against ethanol-induced gastric injury was investigated, as well as was a potential mechanism.

## Results

### Transformation of patchouli alcohol by hydrochloric acid

Figure 1B and C showed the transformation of PA into β-PAE by incubating in 0.01 mol/L hydrochloric acid. Interestingly, 99.95% of PA transformed rapidly after contact with hydrochloric acid, while 60.58% β-PAE was formed. PA converted to β-PAE in a time-dependent fashion, and all PA had been completely transformed within 90 min. This demonstrated that PA mostly converts to β-PAE in agreement with the prediction.

### Transformation of patchouli alcohol by simulated gastric juice

As shown in Fig. 1B and D, PA transformed into β-PAE upon incubation with simulated gastric juice. At 0 min, 2.68% of PA was converted into β-PAE. Over time, the ratio of β-PAE increased in inverse proportion to the decreasing ratio of PA; 70.28% of PA was converted to β-PAE at 120 min. This suggests that PA is mostly converted to β-PAE by simulated gastric juice in a time-dependent manner.

### Transformation of patchouli alcohol by rat gastric juice

The pH of rat gastric juice is 1.5–2.0. Figure 1B and E indicated that PA was converted to β-PAE in rat gastric juice due to its acidic conditions. At first, PA was not transformed into β-PAE. However, the production of β-PAE increased over time and was inversely proportional to the reduction of PA. At 120 min, 61.91% of PA had been converted to β-PAE. The transformation of PA into β-PAE in gastric juice was as expected.

### Effect of PA and β-PAE on GES-1 cells by MTT

**Figure 2:**
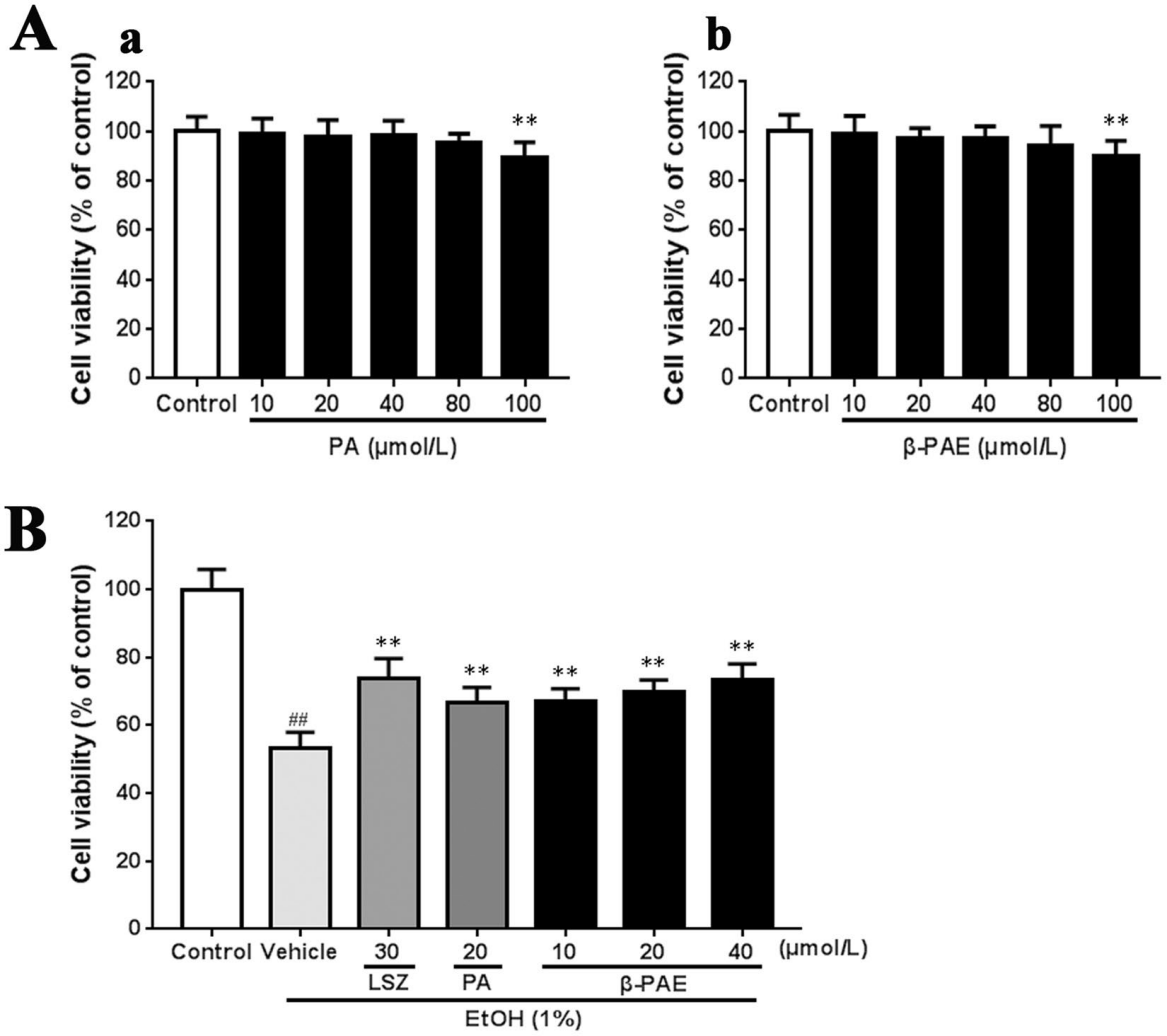
Protective effect of β-PAE against ethanol-induced GES-1 cell death. (**A**) Cytotoxic effects of PA (a) and β-PAE (b) on the GES-1 cell line. (**B**) Effect of β-PAE on cell viability in GES-1 under ethanol-induced cell death. The data are expressed as the mean ± SD and were analysed by ANOVA followed by LSD test. **P < 0.01, *P < 0.05 versus the vehicle or control group. Student’s t-test was performed to compare the control and vehicle groups, ^##^ *P* < 0.01. (EtOH: ethanol, LSZ: lansoprazole).

As shown in Fig. 2A, treatment with PA or β-PAE at the concentration up to 80 μmol/L did not show significant signs of cytotoxicity to GES-1 cells. The concentration from 10 to 40 μmol/L of β-PAE and 20 μmol/L of PA were chosen for the subsequent experiments.

### β-PAE inhibited ethanol induced GES-1 cell death

As shown in Fig. 2B, compared with control group, stimulation with 1% ethanol alone resulted in a marked decrease (53.10 ± 4.88%, *P* < 0.01) in cell viability. In contrast, the cell viabilities in β-PAE (10, 20, 40 μmol/L) groups were significantly increased (66.96 ± 3.88%, 69.81 ± 3.51%, 73.31 ± 4.77%, all *P* < 0.01). The cell viability in PA group was also notably (66.72 ± 4.40%, *P* < 0.01) increased, but of a lesser magnitude than those in β-PAE groups.

### Effect of β-PAE on the gastric mucosal ulcer formation

**Figure 3:**
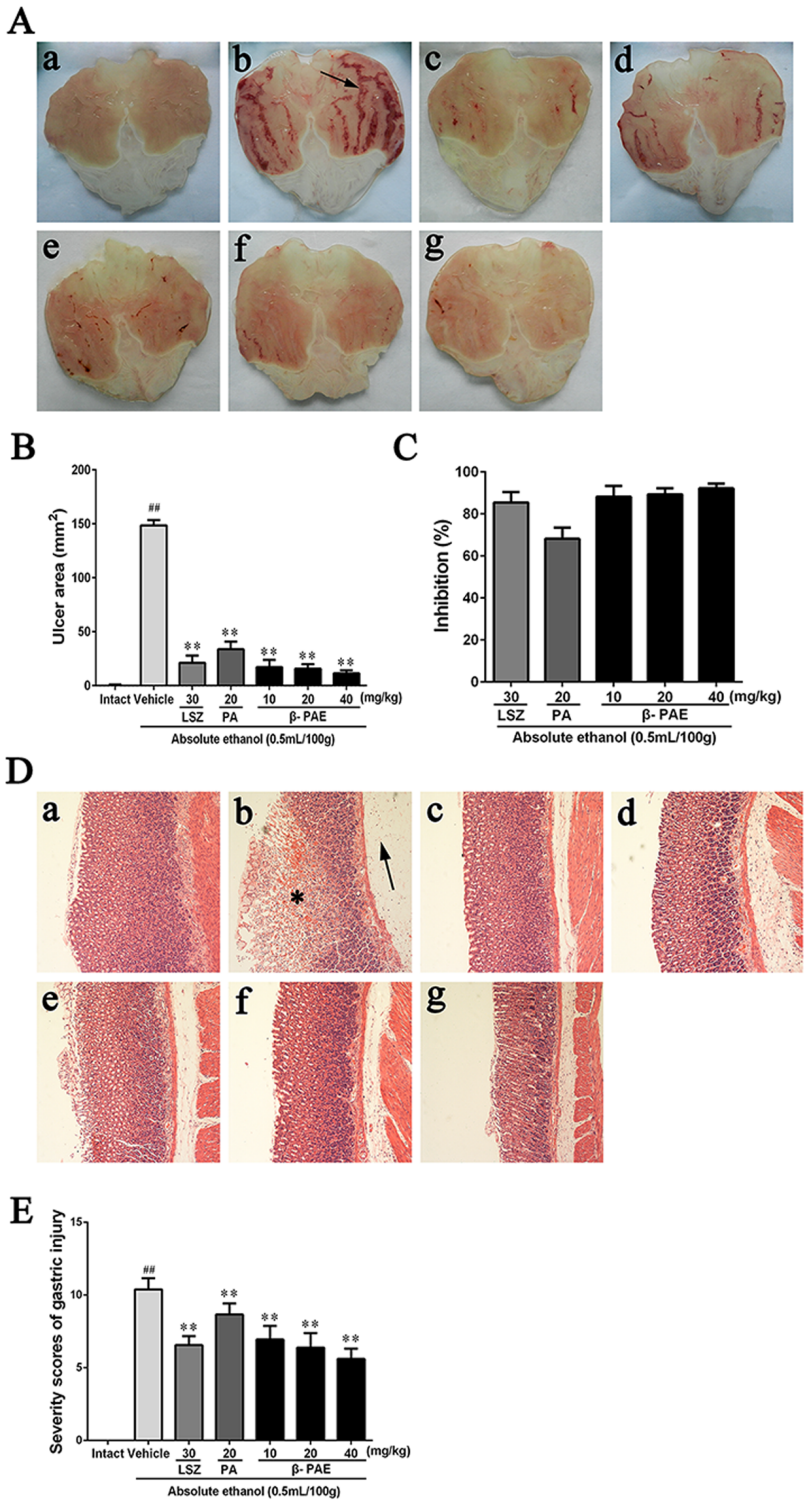
Effects of β-PAE on the macroscopic and microscopic appearance of the gastric mucosa (*n* = 6). (**A**) Macroscopic appearance of the gastric mucosa in seven groups: Intact group (a), Vehicle + Ethanol (b), LSZ (lansoprazole) + Ethanol (c), PA + Ethanol (d), β-PAE (10 mg/kg) + Ethanol (e), β-PAE (20 mg/kg) + Ethanol (f), β-PAE (40 mg/kg) + Ethanol (g). (**B**) Gastric ulcer areas of the gastric mucosa. (**C**) Ulcer inhibition (%). (**D**) Microscopic appearance of the gastric mucosa in seven groups (H&E staining, magnification 100x): Intact group (a); Vehicle + Ethanol (b): *represents disrupted glandular structure on the surface epithelium, and the arrow represents extensive oedema of the submucosal layer; LSZ (lansoprazole) + Ethanol (c); PA + Ethanol (d); β-PAE (10 mg/kg) + Ethanol (e); β-PAE (20 mg/kg) + Ethanol (f); β-PAE (40 mg/kg) + Ethanol (g). (**E**) Total microscopic score. The data are expressed as the mean ± SD and were analysed by ANOVA followed by LSD test. **P < 0.01, *P < 0.05 versus the vehicle group. Student’s t-test was performed to compare the intact and vehicle group, ^##^ *P* < 0.01.

Almost no macroscopic injuries were observed in the intact group (0.07 ± 0.13 mm^2^, Fig. 3Aa). The administration of absolute ethanol (Fig. 3Ab) caused a notable increase (*P* < 0.01) in ulcer area (148.52 ± 12.33 mm^2^) compared with the intact group. This indicated that the ethanol-induced gastric ulcer model was successfully established. Under the effect of lansoprazole (Fig. 3Ac), the ulcer area significantly narrowed (*P* < 0.01), with an average of 21.249 ± 6.67 mm^2^ (85.47% inhibition). After pretreatment with different dosages of β-PAE (10, 20 and 40 mg/kg), the ulcer area was markedly decreased in a dose-dependent manner. Moreover, the minimum ulcer area (11.48 ± 2.80 mm^2^) and the highest inhibition (92.19%) were observed in the β-PAE group (40 mg/kg) (Fig. 3Ag). In the β-PAE group with 20 (Fig. 3Af) and 10 mg/kg (Fig. 3Ae), the ulcer areas were 15.76 ± 4.13 (89.35% inhibition) and 17.27 ± 6.66 mm^2^ (88.22% inhibition), respectively. In contrast, the ulcer area was only 46.95 ± 5.89 (68.15% inhibition) in the PA group (Fig. 3Ad).

### Histological evaluation

Histological observation (Fig. 3D) showed severe harm to the gastric epithelium and oedema of the submucosal layer in the vehicle group (total score: 10.39 ± 0.77). Compared to the vehicle group, the β-PAE group (40 mg/kg, total score: 5.61 ± 0.71) and lansoprazole group (total score: 6.56 ± 0.62) showed better histological appearance of the gastric mucosa, with the least mucosal damage and mildest submucosal oedema. Meanwhile, improved alterations with less mucosal injury and milder oedema were also observed in the β-PAE group at 20 (total score: 6.39 ± 1.00) and 10 mg/kg (total score: 6.94 ± 0.93). In comparison, the histological appearance of the gastric mucosa in the PA group (total score: 8.67 ± 0.76) was the worst among all pre-treated groups. The histological appearance of the stained gastric mucosa was interpreted as a total microscopic score for each group in Fig. 3E.

### Effect of β-PAE on superoxide dismutase (SOD), total glutathione (GSH), catalase (CAT) and malondialdehyde (MDA) levels in gastric tissues

**Figure 4:**
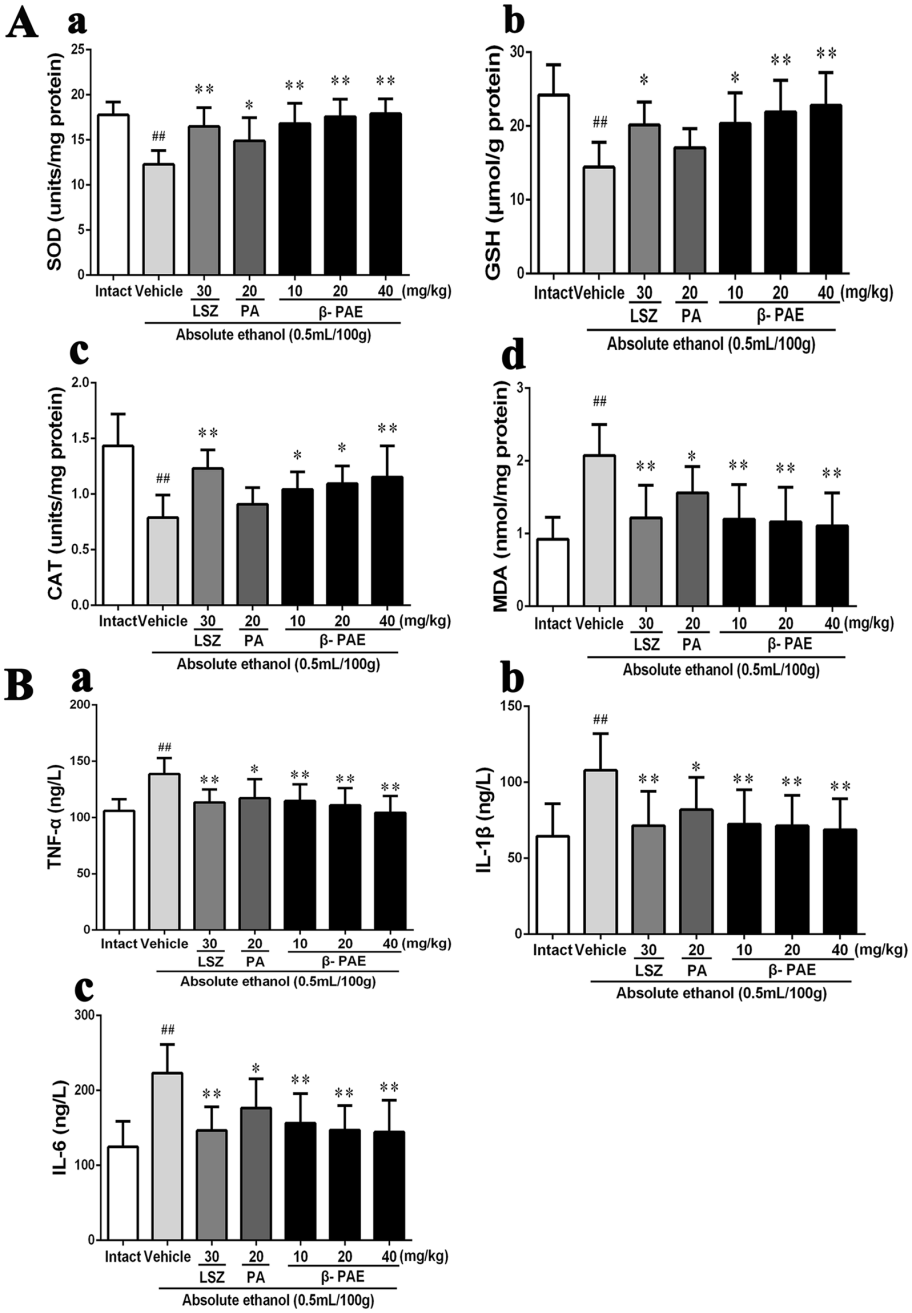
Effect of β-PAE on gastric levels of SOD (**Aa**), GSH (**Ab**), CAT (**Ac**), MDA (**Ad**) and serum levels of TNF-α (**Ba**), IL-1β (**Bb**) and IL-6 (**Bc**) in rats with ethanol-induced gastric injury (*n* = 6). Rats were intragastrically administered lansoprazole (30 mg/kg), PA (20 mg/kg) or β-PAE (10, 20 and 40 mg/kg) prior to intragastric administration of ethanol. The data are expressed as the mean ± SD and were analysed by ANOVA followed by LSD test. **P < 0.01, *P < 0.05 versus the vehicle group. Student’s t-test was performed to compare the intact and vehicle group, ^##^ *P* < 0.01.

As shown in Fig. 4A, absolute ethanol administration inhibited SOD, GSH and CAT activities (*P* < 0.01, *P* < 0.01, *P* < 0.01, respectively) and increased MDA levels (*P* < 0.01). However, after pretreatment with β-PAE at three doses (10, 20 and 40 mg/kg), SOD activity was clearly improved (*P* < 0.01, *P* < 0.01 and *P* < 0.01, respectively), and MDA level was markedly decreased (*P* < 0.01, *P* < 0.01 and *P* < 0.01, respectively) compared to the vehicle group. Meanwhile, CAT activity (*P* < 0.05, *P* < 0.05 and *P* < 0.01, respectively) and GSH level (*P* < 0.05, *P* < 0.01 and *P* < 0.01, respectively) were significantly enhanced in β-PAE groups (10, 20 and 40 mg/kg) compared with those in the vehicle group. In contrast, the effect of PA on SOD and CAT activities, as well as GSH and MDA levels in gastric tissues, was not better than that of β-PAE, and there was no statistical effect on CAT activity (*P* > 0.05) or GSH level (*P* > 0.05).

### Effect of β-PAE on serum cytokines

As shown in Fig. 4B, the serum levels of tumour necrosis factor (TNF)-α, interleukin (IL)-1β and IL-6 were clearly elevated in the vehicle group (*P* < 0.01, *P* < 0.01 and *P* < 0.01, respectively). Nevertheless, pretreatment with three doses of β-PAE (10, 20 and 40 mg/kg) reduced the elevated levels of TNF-α, IL-1β and IL-6 compared with the levels in the vehicle group. The serum levels of TNF-α, IL-1β and IL-6 were also significantly decreased in the PA group compared with those in the vehicle group (*P* < 0.05, *P* < 0.05 and *P* < 0.05, respectively), but the extent of the reduction was less than that seen in the β-PAE pre-treated groups.

### Immunohistochemistry of Fas, FasL and caspase-3 proteins

**Figure 5:**
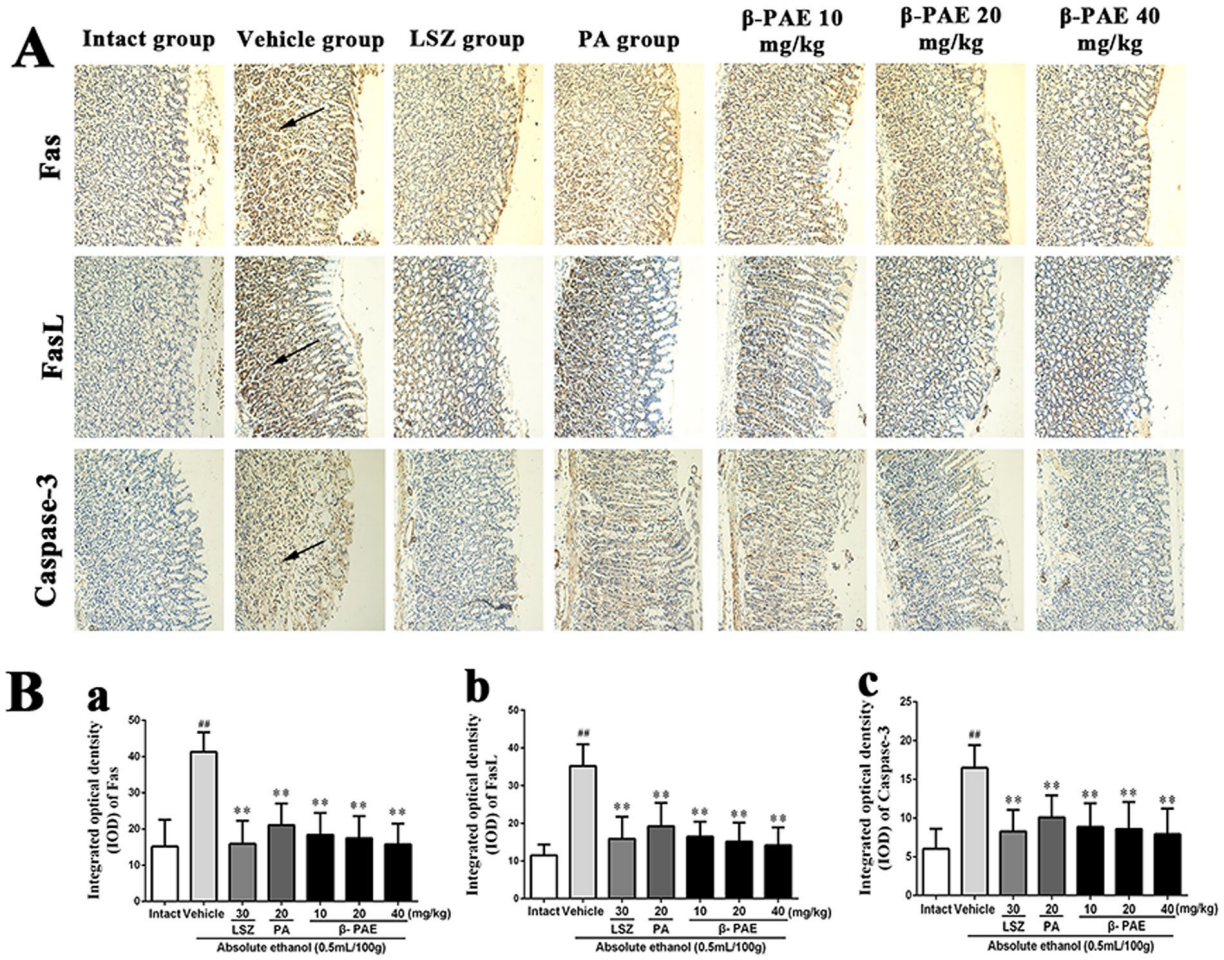
Protein expressions of Fas, FasL and caspase-3 in stomachs of rats (*n* = 6). Rats were intragastrically administered lansoprazole (30 mg/kg), PA (20 mg/kg) or β-PAE (10, 20 and 40 mg/kg) prior to intragastrical administration of ethanol. (**A**) Immunohistochemical staining (magnification 200x), first row: Fas protein; second row: FasL protein; third row: caspase-3 protein. Antigen sites at the arrow. (**B**) Integrated optical density (IOD) of Fas (a), FasL (b) and caspase-3 (c) protein expressions. The data are expressed as the mean ± SD and were analysed by ANOVA followed by LSD test. **P < 0.01, *P < 0.05 versus the vehicle group. Student’s t-test was performed to compare the drug and vehicle groups, ^##^ *P* < 0.01.

The administration of ethanol caused significant up-regulation (*P* < 0.01) of Fas, FasL and caspase-3 proteins compared to the levels in the intact group (IOD: 41.33 ± 5.4, 35.19 ± 5.75, 16.49 ± 2.93, respectively; Fig. 5A and B). The levels of Fas, FasL and caspase-3 proteins were markedly down-regulated (*P* < 0.01) in pre-treated groups (Fas, IOD: lansoprazole, 15.92 ± 6.38; PA, 21.06 ± 5.96; β-PAE, 10 mg/kg, 18.42 ± 6.03, 20 mg/kg, 17.48 ± 6.05, 40 mg/kg, 15.79 ± 5.69. FasL, IOD: lansoprazole, 15.89 ± 5.86; PA, 19.22 ± 6.19; β-PAE, 10 mg/kg, 16.44 ± 3.94, 20 mg/kg, 15.16 ± 5.02, 40 mg/kg, 14.15 ± 4.77. Caspase-3, IOD: lansoprazole, 8.27 ± 2.76; PA, 10.09 ± 2.83; β-PAE, 10 mg/kg, 8.86 ± 3.03, 20 mg/kg, 8.58 ± 3.49, 40 mg/kg, 7.91 ± 3.29.) compared with the levels in the vehicle group. Therefore, β-PAE is better than PA at down-regulating the levels of Fas, FasL and caspase-3 proteins, especially at a dose of 40 mg/kg.

### Effect of β-PAE on the expression of NF-κB signalling-related proteins

**Figure 6:**
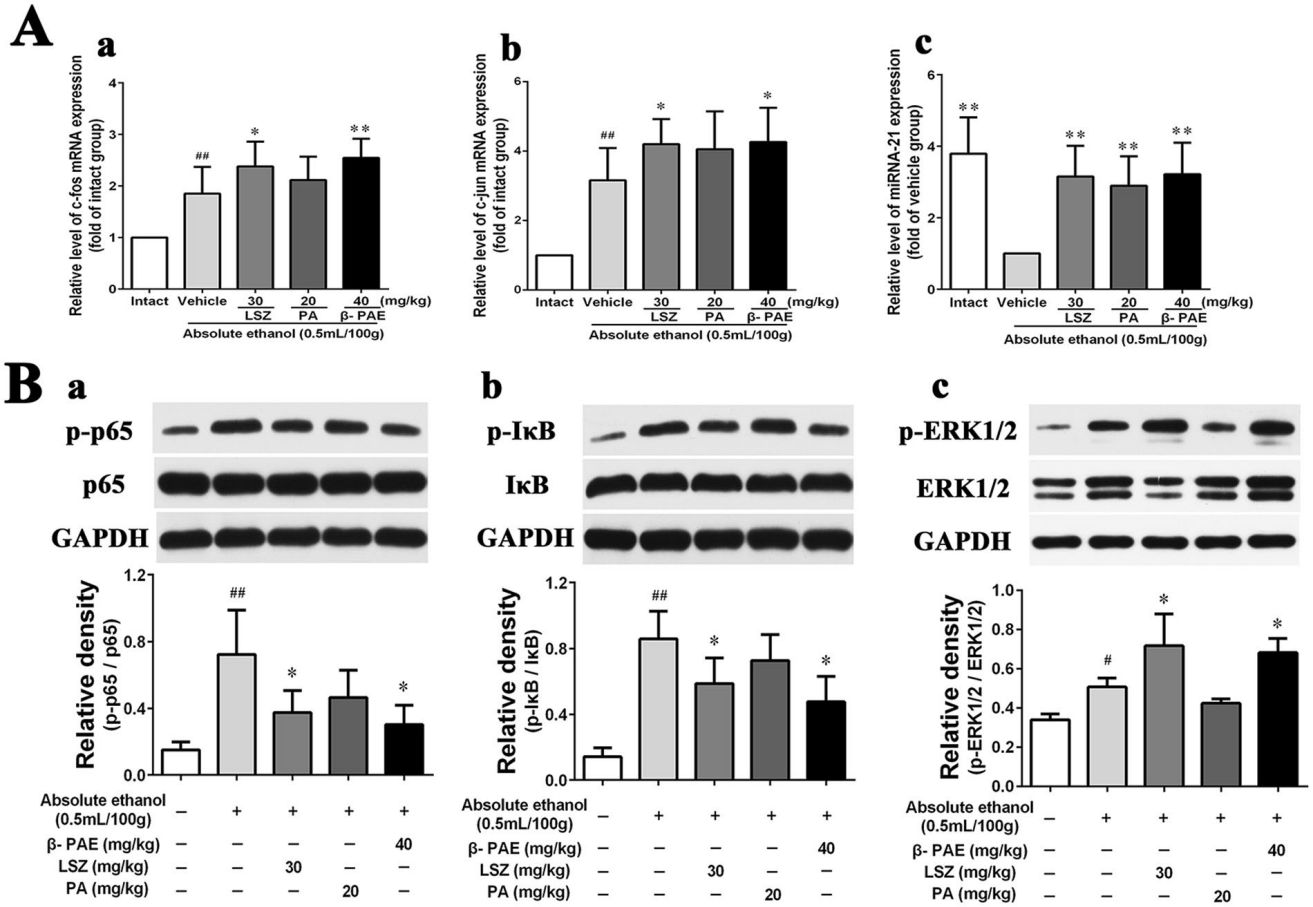
Effect of β-PAE on the mRNA expression of c-fos and c-jun, miRNA expression of miR-21 and expression of NF-κB and ERK1/2 signalling-related proteins in the gastric mucosa of ethanol-induced rats (*n* = 3–5). Rats were intragastrically administered lansoprazole (30 mg/kg), PA (20 mg/kg) or β-PAE (10, 20 and 40 mg/kg) prior to intragastrical administration of ethanol. (**A**) The mRNA expression of c-fos (a) and c-jun (b), and the miRNA expression of miR-21 (c). (**B**) Expression of NF-κB signalling-related proteins (a and b) and ERK1/2 signalling-related proteins (c). Full-length blots/gels are presented in Suppl. Figure S6 and cropping lines are indicated in red color. The data are expressed as the mean ± SD and were analysed by ANOVA followed by LSD test. **P < 0.01, *P < 0.05 versus the vehicle group. Student’s t-test was performed to compare the drug and vehicle groups, ^##^ *P* < 0.01.

The results showed that the ratio between p-p65 and p65 proteins was significantly increased in the vehicle group compared with that in the intact group (*P* < 0.01, Fig. 6Ba), as was the ratio between p-IκB and IκB (*P* < 0.01, Fig. 6Bb). However, β-PAE administration markedly inhibited the increased ratio (*P* < 0.05) and its effect was greater than that for PA, which is consistent with the above antioxidant and anti-inflammatory results. It has been suggested that β-PAE improves antioxidant and anti-inflammatory effect through the NF-κB signalling pathway.

### Effect of β-PAE on the expression of ERK1/2 signalling-related proteins

After oral administration of ethanol, ERK1/2 activation significantly increased in parallel to the intact group (*P* < 0.05, Fig. 6Bc). Compared with the vehicle group, the β-PAE group exhibited a significantly enhanced relative density of p-ERK/ERK (*P* < 0.05) while the PA group did not. Moreover, activated ERK1/2 (phospho-ERK1/2) has been reported to possess an anti-apoptotic effect involved in the repair of gastric mucosal injury. Thus, β-PAE promotes the activation of ERK to trigger healing of the gastric mucosa, in agreement with our immunohistochemistry fingdings of Fas, FasL and caspase-3 proteins.

### Expression of c-fos and c-jun mRNA and miR-21 microRNA in gastric tissues

As shown in Fig. 6A, oral administration of ethanol induced up-regulation of c-fos and c-jun expression (1.85-fold and 3.16-fold, respectively) and down-regulation of miR-21 expression (1.00-fold). c-fos and c-jun mRNA expressions was markedly increased in the lansoprazole group (2.38-fold and 4.20-fold, respectively), PA group (2.12-fold and 4.06-fold, respectively) and β-PAE group (2.55-fold and 4.26-fold, respectively) compared with the expression levels in the vehicle group. Additionally, administration of lansoprazole (3.16-fold), PA (2.90-fold) or β-PAE (3.22-fold) significantly restored the ethanol-induced depletion of miR-21. Thus, β-PAE pretreatment resulted in the highest expression of miR-21. Therefore, β-PAE up-regulates the expressions of c-fos and c-jun mRNA, as well as miR-21 microRNA.

## Discussion

Despite its wide applications in flavouring, Pogostemonis Herba, which is rich in PA, possesses protective pharmacological activity^11^. Our previous study reported that PA has a gastroprotective effect against experimental gastric injury, but the gastric metabolism of PA and the anti-ulcer properties of its metabolite remained unclear. In this study, the metabolism of PA was first elucidated in the gastric juice of rats. Moreover, it is the first report of the gastroprotective effect of β-PAE and its underlying mechanism against ethanol-induced gastric injury.

Oral ingestion is the most common mode of administration of drugs, including PA. After oral delivery, PA enters the stomach and is metabolized. PA is chemically modified under acidic conditions, and is mostly transformed into β-PAE^10^. Our results confirmed that most PA rapidly converts into β-PAE in hydrochloric acid. Gastric juice, a digestive fluid formed in the stomach, is composed of hydrochloric acid, water, digestive enzymes, mucus, electrolytes and an intrinsic factor^12^. The pH of gastric juice is 1.5 to 3.5. Because of its acidity, PA was hypothesized to be chemically modified in gastric juice. In our findings, PA was affirmed transformed into β-PAE in simulated gastric juice as well as in rat gastric juice in a time-dependent manner *in vitro*. Moreover, PA was also affirmed to transform β-PAE *in vivo* (Supplementary Information). The transformation mechanism of PA into β-PAE may be associated with a series of Wagner-Meerwein shifts in gastric juice. The gastric transformation of PA into β-PAE after oral administration was previously hypothesized. Additionally, the experiments on the relationship between the pH and the transformation from PA to β-PAE showed that the pH of gastric juice was 2.43 ± 0.32 in ethanol-induced rats and 7.42% of PA was transformed into β-PAE in simulated gastric juice at pH 2.5. This meant that PA was perhaps not effectively transformed into β-PAE in atrophic gastritis (Supplementary Information).

Reactive oxygen species (ROS), i.e., chemically reactive species containing oxygen, play a crucial role in the pathogenesis of gastric injury. Ethanol consumption can significantly stimulate ROS generation and promote lipid peroxidation followed by serious damage to the gastric mucosa. The gastrointestinal tract has enzymatic and non-enzymatic defence barriers against ROS-activated lipid peroxidation^8^. SOD, an important intracellular enzymatic antioxidant, constitutes the first line of defence against ROS. It catalyses superoxide radicals into more stable hydrogen peroxide to protect cells from the toxic effect of ROS^1^. Unsaturated fatty acids are attacked by ROS in the biofilm system, and are converted into MDA through lipid peroxidation. Thus, MDA can be used to quantify and identify lipid peroxidation^13^. GSH is also an important intracellular antioxidant that can effectively reflect the oxidative stress state^14^. CAT can scavenge ROS by catalysing the decomposition of H_2_O_2_ into water and oxygen^15^. In the present study, ethanol consumption led to significantly decreased contents of SOD, GSH and CAT and increased contents of MDA in gastric tissues. However, β-PAE pretreatment clearly restored the reductive contents of SOD, GSH and CAT and attenuated the increase in MDA level. The results suggested that β-PAE was better than PA at inhibiting oxidative gastric injury, which explains its stronger gastroprotective effect.

Under the effect of various damaging factors, many inflammatory cytokines were generated, resulting in the infiltration of neutrophils and subsequent lesions in the gastric mucosa. The levels of several pro-inflammatory cytokines, such as TNF-α, IL-1β and IL-6, were reported to significantly increase in gastric tissue and serum after ethanol administration^16^. TNF-α, a potent stimulator of neutrophil infiltration, can activate the neutrophils to greatly accumulate around the ulcer. The accumulated neutrophils can lead to gastric microcirculatory disturbance and aggravate the formation of gastric mucosal ulcers. TNF-α induces cell growth and differentiation at low concentrations, but also promotes the secretion of IL-1, IL-6 and other cytokines to cause inflammation by activating leucocytes at high concentrations^17^. Increased levels of IL-6 activate neutrophils and macrophages at the inflammatory site to trigger oxidative stress and lysosomal enzymes responsible for gastric mucosal lesions^4^. Additionally, IL-1β is involved in the evolution of ethanol-induced gastric injury and the recruitment of other cytokines such as TNF-α and prostaglandins^18^. Similarly, our experimental results showed that the levels of TNF-α, IL-1β and IL-6 clearly increased in the serum after ethanol administration. However, β-PAE pretreatment significantly reduced the ethanol-induced increases in TNF-α, IL-1β and IL-6, better than the effect of PA. Inhibition of inflammation may be an important mechanism contributing to the gastroprotective effect of β-PAE against ethanol-induced gastric ulcer.

The interaction between apoptosis and proliferation determines the formation of gastric ulcer. Apoptosis is a process of programmed cell death that has been implicated in ethanol-induced gastric mucosal injury^19^. After gastric mucosal damage, up-regulated expression of Fas, FasL and caspase-3 triggers apoptosis which is an important mechanism of ulcer formation. Fas and FasL are expressed in gastric mucosal cells and are generated as tripolymers to induce apoptosis. Fas is a transmembrane protein that mainly occurs in the membrane receptor form and regulates the function of FasL. FasL can promote the apoptosis of gastric mucosal cells through autocrine and paracrine modes of action^20, 21^. Caspase is a protease that can be divided into initial and effective forms according to their different functional characteristics in apoptotic signal transduction. Caspase-3 is an indispensable protease for the apoptosis protease cascade reaction and its expression leads to cell death^16^. The Fas-associated death domain interacts with the death effector domain of caspase-8. Then caspase-8 activates itself through the formation of oligomers to stimulate the activation of caspase-3, which leads to apoptosis. Thus caspase-3 is associated with Fas and FasL in apoptosis^22^. As in previous studies, our results showed that Fas, FasL and caspase-3 expression significantly increased in the gastric mucosa of rats after ethanol administration. However, the expression of Fas, FasL and caspase-3 was inhibited in the drug groups, and β-PAE was much more effective than PA in reducing Fas, FasL and caspase-3 expression. We hypothesize that the suppression of Fas, FasL and caspase-3 biosynthesis may perform an important role in enhancing the protective effect of β-PAE against ethanol-induced gastric ulcer.

Furthermore, the mechanism underlying the protective effect of β-PAE against ethanol-induced gastric mucosal injury is multifactorial. It has been demonstrated that NF-κB is an ideal mediator of pro-inflammatory molecule expression in gastric mucosal lesions. In the resting state, NF-κB is located in the cytosol as a p50/p65 protein polymer bound to the IκB inhibitor. The upstream signal activates IκB kinase to phosphorylate through a cascade reaction. Additionally, the p65 subunit is phosphorylated followed by translocation to the nucleus. In this way, NF-κB promotes the expression of corresponding inflammation-related genes, such as TNF-α, IL-1β and IL-6 to induce inflammation. Cytokine secretion leads to mitochondrial ROS generation enhances the effect of oxidative stress^23^. Our results confirm that β-PAE pretreatment can inhibit the NF-κB signalling pathway and subsequently improve the protective effect against oxidative stress and inflammatory stimuli-induced gastric ulceration.

Alcohol-induced gastric mucosal injury is a programmed damage response. Epithelial defects on the gastric mucosal surface leads to superficial mucosal injury, and subsequent damage is further developed with mucosal ischemia, hypoxia and necrosis through microvascular endothelial cell injury, ultimately resulting in deep mucosal^24^. It has been reported that activated ERK 1/2 can promote the proliferation of gastric mucosal cells and the repair of the gastric mucosa. In the healing process of gastric ulceration in rats, ERK 1/2 activity is significantly enhanced, and the healing process can be delayed if the ERK 1/2 signalling pathway is blocked. Phosphorylated ERK 1/2 is the active form of ERK 1/2^25^. Activated ERK 1/2 can regulate the expression of activator protein 1 (AP-1) to promote the proliferation of gastric mucosal cells in rats^26^. AP-1 is an intracellular transcription activator composed of c-Fos and c-Jun family members. Increased expression of c-fos and c-jun mRNA can accelerate the repair of gastric mucosal lesions. In addition, AP-1 can activate miR-21 transcription^27^. Up-regulated expression of miR-21 can inhibit alcohol induced apoptosis in human gastric epithelial cells^28^. Similarly, our results demonstrated that β-PAE not only enhanced ERK 1/2 activity, but also enhanced c-fos and c-jun mRNA and miR-21 microRNA expressions. Thus, the gastroprotective effect of β-PAE against ethanol-induced gastric ulcer may be associated with activation of ERK 1/2 signaling pathway and anti-apoptotic activity.

In conclusion, our findings clearly demonstrate that PA transforms into β-PAE in gastric juice, subsequently, β-PAE possesses a better gastroprotective effect than PA against ethanol-induced gastric injury. β-PAE significantly narrowed the gastric ulcer areas and submucosal oedema in a dose-dependent manner. The potential mechanisms may be attributed to the inhibition of oxidative damage and inflammatory responses, as well as the activation of anti-apoptotic activity. Therefore, β-PAE exerts a gastroprotective effect, and has the potential to be a functional food additive.

## Materials and Methods

### Animals

Male Sprague Dawley (SD) rats (180–220 g) were obtained from the Laboratory Animal Center of the Guangzhou University of Chinese Medicine (Guangzhou, China). The rats were maintained in an environmentally controlled condition (22 ± 2 °C, relative humidity of 50 ± 5%) and fed with standard forage and clean water *ad libitum*. All protocols were approved by the Animal Experimental Ethics Committee of Guangzhou University of Chinese Medicine (Guangzhou, China, No. 2016047) and conducted in accordance with the National Institutes of Health (NIH) guidelines.

## Reagents

PA and β-PAE were isolated from patchouli oil according to published articles^29, 30^. Lansoprazole (purity ≥98%) and ethanol (purity ≥99.7%) were purchased from the Aladdin Industrial Corporation (Shanghai, China). Antibodies for p65, phosphor-p65, Fas and Fas ligand were obtained from Abcam plc. (Cambridge, UK). Antibodies for IκB, phosphor-IκB and caspase-3 were purchased from Santa Cruz Biotechnology Inc. (Santa Cruz, CA, USA). Antibodies for extracellular signal-regulated kinase1/2 (ERK1/2), phospho-ERK1/2, and GAPDH were obtained from Cell Signaling Technology Inc. (Danvers, MA, USA). All other chemicals and reagents were of commercially available analytical grade.

### Preparation of simulated gastric juice and rat gastric juice

Simulated gastric juice was prepared as previously described, with slight modifications^31^. To prepare the simulated gastric juice, pepsin was dissolved in sterile saline (0.9% sodium chloride) to obtain a final concentration of 3.2 g/L pepsin solution. The pH of the pepsin solution was adjusted to 2 with 1 mol/L hydrochloric acid using a pH meter. Rat gastric juice was obtained through pylorus ligature for 3 h^9^.

### Transformation of patchouli alcohol by hydrochloric acid

The PA sample (1 mg/mL) was obtained by dissolving PA in 0.1% Tween 80 solution. Fifteen mL hydrochloric acid (0.01 mol/L) was poured into a conical flask with a stopper. Then, six identical conical flasks were incubated on a shaker at 90 rpm for 1 h. Next, 3 mL of each PA sample was added to the conical flasks simultaneously and the negative control group contained 0.1% Tween 80 solution. One conical flask was removed at 0, 30, 60, 90 and 120 min. The mixture in each flask was extracted with 3 mL n-hexane, and the organic layer was dried with anhydrous sodium sulphate. Finally, 1 mL organic extracts was subjected to GC-MS analysis. The experiment was repeated three times.

### Transformation of patchouli alcohol by simulated gastric juice

The PA sample was prepared as described above. Fifteen mL of simulated gastric juice was poured into a conical flask with a stopper. Six identical conical flasks were incubated on a shaker at 90 rpm in a humidified atmosphere (37 °C, 95% air and 5% CO_2_) for 1 h. Then, 3 mL of PA sample was added to each conical flasks simultaneously except the negative control group, and one conical flask was removed at 0, 30, 60, 90 and 120 min. After removal, the mixture was extracted with 3 mL n-hexane, and the organic layer was dried with anhydrous sodium sulphate. Finally, 1 mL of organic extracts was subjected to GC-MS analysis. The experiment was repeated three times.

### Transformation of patchouli alcohol by rat gastric juice

The PA sample was prepared as described above. Three mL of rat gastric juice was poured into conical flasks with a stopper. Then, six identical conical flasks were incubated on a shaker at 90 rpm in a humidified atmosphere for 1 h. Next, 0.6 mL of PA sample was added to the conical flasks simultaneously, except for the negative control group and one conical flask was removed at 0, 30, 60, 90 and 120 min. The mixture was extracted with 3 mL n-hexane, and the organic layer was dried with anhydrous sodium sulphate. Finally, 1 mL of organic extracts was subjected to GC-MS analysis. The experiment was repeated three times.

### GC–MS analyses

The metabolites of PA in hydrochloric acid and simulated or rat gastric juice were analysed using an Agilent Technologies GC7890A-MSD5975C system equipped with an auto sampler. Chromatographic separation was achieved with an HP-5MS 5% Phenyl Methyl Silox column (30 m × 250 μm × 0.25 μm). Helium (purity of 99.999%) was used as the carrier gas at a flow rate of 1.3 mL/min, and the injector split was 1:60. The injector temperature was 250 °C and the column oven temperature was programmed from 50 °C (held for 2 min), increased to 120 °C with a rate of 60 °C/min, and was then isothermal for 5 min. Then, the temperature increased to 150 °C at 10 °C/min, followed by a 60 °C/min increase to 235 °C and was maintained at 235 °C for 15 min. Full scan data were obtained from 35 *m/z* to 400 *m/z*.

### Cell culture and treatments

Human GES-1 cells were kindly provided by the First Affiliated Hospital of Guangzhou University of Chinese Medicine. They were cultured in high glucose DMEM supplemented with 10% (v/v) heat-inactivated FBS at 37 °C in humidified atmosphere of 95% air and 5% CO_2_.

PA or β-PAE was dissolved in dimethyl sulfoxide (DMSO), the final concentration of DMSO in the test solutions was less than 0.1%. The GES-1 cells grown in the medium containing an equivalent amount of DMSO without other drugs were used as control.

To study the cytotoxicites of PA and β-PAE, GES-1 cells were divided into control group (without PA, β-PAE treatment), PA groups (10, 20, 40, 80, 100 μmol/L), and β-PAE groups (10, 20, 40, 80, 100 μmol/L).

To investigate the possible protective effect of PA and β-PAE, GES-1 cells were divided into control group (without PA, β-PAE and 1% ethanol treatment), Vehicle group (only 1% ethanol for 24 h), ethanol plus lansoprazole (30 μmol/L) group, ethanol plus PA (20 μmol/L) group, and ethanol plus β-PAE (10, 20, 40 μmol/L) groups. For ethanol plus drug (lansoprazole, PA or β-PAE) groups, GES-1 cells were pre-treated with drug for 4 h prior to co-cultivation with 1% ethanol for 24 h. The modeling concentration of ethanol was selected based on the results of our preliminary experiment (Supplementary Information).

### Cell viability assay

Cell viability was measured by the MTT assay as previously described^32^. Briefly, after drug treatment, 20 μL MTT solution (final concentration, 1 mg/mL) was added to each well for an additional 4 h at 37 °C. The supernatants were then aspirated off and, formazan crystals were dissolved with 150 μL of DMSO. Subsequently, the optical density was determined at 490 nm using a FLUOstar Optima microplate reader (BMG Labtech, Offenbury, Germany). Cell viability was expressed as percentage of the non-treated control.

### Administration and dose selection in a gastroprotective experiment

Male Sprague Dawley rats were allowed to acclimatize to the conditions of laboratory for 7 days in the pre-experimental period. All rats were randomly divided into seven groups of six animals each. The intact and vehicle groups received (i.g.) 0.1% Tween 80 throughout the course of the experiment. The pretreatment groups received (i.g.) lansoprazole (30 mg/kg), PA (20 mg/kg) and various doses of β-PAE (10, 20 and 40 mg/kg) dissolved in 0.1% Tween 80 as described in the article for a period of 7 days. All rats were intragastrically administered once daily. The dose selection of β-PAE was based on a preliminary dose responsive experiment on ethanol-induced gastric injury.

### Ethanol-induced gastric injury

All rats were fasted, with access only to clean water for 24 h prior to experimentation. One h after the last administration, rats were administered with absolute ethanol by oral probe (0.5 mL/100 g) to induce acute gastric injury; those in the intact group received water. Blood samples for each rat were collected after one h, at which point all rats were euthanized and their stomachs were rapidly removed, opened and cleaned. Subsequently, stomach tissues were photographed, and the ulcer area was analysed using ImageJ (1.47 v, National Institutes of Health, USA). The inhibition percentage was calculated using the following formula: Inhibition percentage = [(UAcontrol − UAtreated)/UAcontrol] × 100%. After photographing, 0.5 cm × 0.5 cm of gastric tissue was obtained from each stomach and immersed in 4% paraformaldehyde for histological and immunohistochemical evaluation. Serum was collected by centrifuging blood samples at 740 × g for 10 min. The serum and the rest of gastric tissue were stored at −80 °C for biochemical analyses.

### Histopathological assessment

Gastric tissues fixed in 4% paraformaldehyde were dehydrated and embedded in paraffin. Five-μm thick sections were sliced, deparaffinized and stained with hematoxylin–eosin (H&E). Evaluation was performed under a light microscope by three pathologists who were blinded to this study. According to the criteria as previously reported, samples were quantified according to a scoring system: epithelial cell loss was scored from 0 to 3, oedema in the submucosa was scored from 0 to 4, haemorrhagic damage was scored from 0 to 4, and the presence of inflammatory cells was scored from 0 to 3. Summation of the four partial scores gave the total microscopic score.

### Measurement of superoxide dismutase (SOD), total glutathione (GSH), catalase (CAT) and malondialdehyde (MDA) activity

Tissues stored at −80 °C were thawed and homogenized in homogenization Tris-buffer (20 mM, pH = 7.5) on ice using a homogenizer (T10 Basic Ultra Turrax, IKA, Germany). The homogenates were centrifuged at 12,000 × g and 4 °C for 10 min. The supernatants were used to determine the activities of SOD, CAT, GSH and MDA. First, the protein concentration of supernatants was measured using a Bradford Protein Assay Kit. SOD, CAT, GSH and MDA levels were obtained using commercial assay kits according to the manufacturer’s instructions (Jiancheng Company, Nanjing, China).

### Determination of cytokines

The levels of cytokines in the serum, including TNF-α, IL-1β and IL-6, were measured using commercial enzyme-linked immunosorbent assay (ELISA) kits (eBioscience, USA) according to the manufacturer’s instructions.

### Immunohistochemical evaluation

Paraffin sections were prepared as described above. The sections were washed with phosphate-buffered saline, blocked with 10% bovine serum albumin and incubated with anti-Fas antibodies (dilution 1:400), anti-Fas ligand antibodies (dilution 1:400) and anti-Caspase-3 antibodies (dilution 1:100). Sections were incubated with goat anti-rabbit secondary antibody and labelled with horseradish peroxidase at 37 °C for 30 min. The slides were rewashed and stained with 3-3′-diaminobenzidine, followed by redyeing with haematoxylin. The sections were observed under a light microscope and photographed. The integrated optical density (IOD) of each photograph was analysed using Image-Pro Plus 6.0 (Media Cybernetics, Inc.).

### Western blot analysis

Gastric tissues were minced and homogenized in PBS on ice and lysed in RIPA buffer with protease and phosphatase inhibitors for 30 min on ice. Thereafter supernatants were obtained through centrifugation at 12,000 × g and 4 °C for 10 min and protein concentrations were determined using the BCA Protein Assay kit following the manufacturer’s instructions. Protein samples were separated by SDS-PAGE gels and transferred to PVDF membranes. The membranes were blocked with 5% (w/v) skim milk in TBST and incubated with the appropriate primary and secondary antibodies. Working dilution were prepared as follows: p65 (1:1000), phospho-p65 (1:1000), IκB (1:500), phospho-IκB (1:500), ERK1/2 (1:1000), phosphor-ERK1/2 (1:1000) and GAPDH (1:1000). Protein bands were detected using an ECL Advanced kit (Amersham Biosciences, Buckinghamshire, UK). A GAPDH antibody was used to verify equal loading of samples.

### Determination of c-fos and c-jun mRNA and miR-21 microRNA by qRT-PCR

Total RNA was extracted from specimens with TRIzol reagent (Invitrogen). The purity of RNA was evaluated by determining the ratio of absorbance at 260 nm to that at 280 nm, and the ratio was between 1.8 and 2.0. The level of c-fos, c-jun and miR-21 in each sample was measured using FastStart Universal SYBR Green Master (Rox) and a RevertAid First Strand cDNA Synthesis Kit according to the protocol provided by the manufacturer. β-actin acted as an internal control for the relative quantification of c-fos and c-jun, and U6 acted as an internal control for the relative quantification of miR-21. Primer sequences are listed in Table 1. PCR amplification was performed as follows: pre-denaturation at 95 °C for 10 min, followed by 40 cycles of 95 °C for 15 s and 60 °C for 20 s. The relative quantification of gene expression was calculated using the 2^−ΔΔCt^ method.

**Table 1:** Primer sequences.

Gene	Orientation	Sequence (5′ to 3′)
c-fos	Forward	GCC TCG TTC CTC CAG TCC GA
	Reverse	TGC GAT GGA AAG GCC AGC CC
c-jun	Forward	CAG GTG GCA CAG CTT AAA CA
	Reverse	CGC AAC CAG TCA AGT TCT CA
β-actin	Forward	GCC AGA GCG GGA GTG GTG AA
	Reverse	GGC TTG GGC TCA GGG TCA TT
miR-21	Stem-loop	CTC AAC TGG TGT CGT GGA GTC GGC AAT TCA GTT GAG TCA ACA TC
	Forward	ACA CTC CAG CTG GGT AGC TTA TCA GAC TGA
	Reverse	CTC AAC TGG TGT CGT GGA
U6	Forward	CTC GCT TCG GCA GCA CA
	Reverse	AAC GCT TCA CGA ATT TGC GT

### Statistical analysis

All data are expressed as the mean ± SD, and a value of *P* < 0.05 was considered statistically significant. Statistical analyses were performed using a one-way analysis of variance (ANOVA) followed by an LSD test for multiple comparisons using SPSS software 20.0. Student’s t-test was used to evaluate the comparison between two groups.

## Electronic supplementary material

Supplementary information
