# Supplementary material for: Transformation of patchouli alcohol to β-patchoulene by gastric juice: β-patchoulene is more effective in preventing ethanol-induced gastric injury
Source: Sci Rep. 2017 Jul 17;7:5591. doi: 10.1038/s41598-017-05996-5 (PMC5514077; doi:10.1038/s41598-017-05996-5)

# Supplementary information

## Transformation of patchouli alcohol to β-patchoulene by gastric juice: β-patchoulene is more effective in preventing ethanol-induced gastric injury

**Yuhong Liu[1,+], Jiali Liang[1,+], Jiazhen Wu[2], Hanbin Chen[2], Zhenbiao Zhang[1], Hongmei Yang[1], Liping Chen[5], Haiming Chen[3] , Ziren Su[1,4,*], and Yucui Li[1,*]**

[1]Guangdong Provincial Key Laboratory of New Drug Development and Research of Chinese Medicine, School of Chinese Materia Medica, Guangzhou University of Chinese Medicine, Guangzhou, 510006, China

[2]The First Affiliated Hospital of Chinese Medicine, Guangzhou University of Chinese Medicine, Guangzhou, 510405, China

[3]Guangdong Provincial Hospital of Chinese Medicine, Guangzhou University of Chinese Medicine, Guangzhou, 510120, China

[4]Dongguan Mathematical Engineering Academy of Chinese Medicine, Guangzhou University of Chinese Medicine, Dongguan, 523808, China

[5]College of Pharmacy, Jinan University, Guangzhou, 510632, China

**[*]Correspondence and requests for materials should be addressed to Y.L. (liyucui@gzucm.edu.cn) or Z.S. (email: suziren@126.com)**

[+]These authors contributed equally to this work

**Gastric metabolism of patchouli alcohol *in vivo***

Male Sprague Dawley rats were divided into five groups of seven animals each. Each group had a negative control rat without patchouli alcohol (PA) pre-treatment. All rats were received (i.g.) PA solution (20 mg/kg) except negative control rat. Subsequently, five groups of rats were euthanized at 0, 30, 60, 90 and 120 min, respectively. Then their stomachs were rapidly removed with ligation and opened, washed with saline until the volume was 5 mL. The mixture was extracted with 3 mL n-hexane, and the organic layer was dried with anhydrous sodium sulphate. Finally, 1 mL of organic extracts was subjected to GC-MS analysis.

As shown in Suppl. Figure S1, PA was really converted to β-patchoulene (β-PAE) *in vivo*. At first, no β-PAE occurred. Then PA began to transform into β-PAE at 60 min. At 90 and 120 min, 15.00% and 14.78% of PA had been converted to β-PAE, respectively. The transformation of PA into β-PAE *in vivo* was as expected, and the percent conversion was low. Therefore, the low conversion may result in the clear disparity in the comparison of the results obtained for the activity of PA and β-PAE in the animal experiments.

**Preliminary experiment for the administration time**

Male Sprague Dawley rats were divided into ten groups of six animals each. From day 1 to day 9, the rats from intact and vehicle group received (i.g.) vehicle (0.1 % Tween 80). In contrast, 9-day group received (i.g.) PA or β-PAE at the same dosage of 20 mg/kg (the dose selection were based on our previous studies[1,2]) from day 1 to day 9. One day later, the rats from 7-day group began to receive (i.g.) PA or

β-PAE for 7 days. Additionally, the rats from 5-day group received (i.g.) PA or β-PAE from day 5 to day 9. On the sixth day, the rats from 3-day group began to receive (i.g.) PA or β-PAE for 3 days. In PA or β-PAE pre-treatment groups, all rats received (i.g.) vehicle (0.1 % Tween 80) at other times. All rats were intragastrically administered once daily. Experimental procedure was shown in Suppl. Fig. S2.

All rats were fasted, with access only to clean water for 24 h prior to experimentation. One hour after the last administration, rats orally received with absolute ethanol (0.5 mL/100g) to induce acute gastric injury; those in the intact group received water. All rats were euthanized and their stomachs were rapidly removed, opened and cleaned. Subsequently, stomach tissues were photographed, and the ulcer area was analysed using ImageJ (1.47v, National Institutes of Health, USA). The inhibition percentage was calculated using the following formula: Inhibition percentage = [(UAcontrol – UAtreated) / UAcontrol] × 100%.

As shown in Suppl. Figure S3, administration of absolute ethanol (Suppl. Fig. S3Ab) caused a significant increase ($P$<0.01) in ulcer area (244.15 ± 4.40 $mm^2$) compared with that in the intact group. In contrast, the ulcer areas in PA and β-PAE pre-treatment groups were marked lower (all $P$<0.01). Moreover, in PA pre-treatment groups, the minimum ulcer area (16.44 ± 3.96 $mm^2$) and the highest inhibition (93.27 ± 1.62 %) were observed in 7-day group (Suppl. Fig. S3Ae). In β-PAE pre-treatment groups, the minimum ulcer area (8.19 ± 4.49 $mm^2$) and the highest inhibition (96.65 ± 1.83 %) were also observed in 7-day group (Suppl. Fig. S3Ai).

In 3-day (Suppl. Fig. S3Ac), 5-day (Suppl. Fig. S3Ad) and 9-day (Suppl. Fig.

S3Af) groups of PA pre-treatment, the ulcer areas were 94.05 ± 3.72 (61.48 ± 1.10 % inhibition), 37.53 ± 3.76 (84.62 ± 1.65 % inhibition) and 15.80 ± 5.59 $mm^2$ (93.54 ± 2.23 % inhibition), respectively. Additionally, in 3-day (Suppl. Fig. S3Ag), 5-day (Suppl. Fig. S3Ah) and 9-day (Suppl. Fig. S3Aj) groups of β-PAE pre-treatment, the ulcer areas were 53.06 ± 7.58 (78.25 ± 3.23 % inhibition), 20.60 ± 5.31 (91.57 ± 2.11 % inhibition) and 9.18 ± 5.18 $mm^2$ (96.24 ± 2.12 % inhibition), respectively.

In PA or β-PAE pre-treatment groups, the ulcer area in 9-day group did not show superior anti-ulcerogenic potential than those in the 7-day group. Meanwhile, neither 3-day group nor 5-day group significantly protect gastric mucosa against the damage of ethanol by observation of ulcer area. Therefore, a period of 7 days may be best time for PA and β-PAE to function best on the mucous in rats.

**Preliminary experiment for the dose selection**

The dose selection of PA was based on the published article[1]. To study the dose selection of β-PAE, all rats were randomly divided into seven groups of six animals each. The intact and vehicle groups received (i.g.) 0.1 % Tween 80 throughout the course of the experiment. The pretreatment groups received (i.g.) various doses of β-PAE (5, 10, 20, 40 and 60 mg/kg) dissolved in 0.1 % Tween 80 as described in the article for a period of 7 days. All rats were intragastrically administered once daily. All rats were fasted, with access only to clean water for 24 h prior to experimentation. One hour after the last administration, rats orally received with absolute ethanol (0.5 mL/100g) to induce acute gastric injury; those in the intact group received water. All rats were euthanized and their stomachs were rapidly removed, opened and cleaned.

Subsequently, stomach tissues were photographed, and the ulcer area was analysed using ImageJ (1.47v, National Institutes of Health, USA). The inhibition percentage was calculated using the following formula: Inhibition percentage = [(UAcontrol – UAtreated) / UAcontrol] × 100%.

As shown in Suppl. Figure S3, administration of absolute ethanol (Suppl. Fig. S3Db) caused a significant increase ($P$<0.01) in the ulcer area (138.44 ± 4.77 $mm^2$) compared with that in the intact group. By contrast, the ulcer area in β-PAE pre-treatment groups was marked lower (all $P$<0.01). Moreover, the minimum ulcer area (8.59 ± 3.79 mm2) and the highest inhibition (93.71 ± 2.94 %) were observed in the 40 mg/kg group (Suppl. Fig. S3Df). In the β-PAE group with 5 (Suppl. Fig. S3Dc) and 60 mg/kg (Suppl. Fig. S3Dg), the ulcer areas were 23.78 ± 5.04 (82.72 ± 4.17 % inhibition) and 8.96 ± 3.68 mm2 (93.45 ± 2.87 % inhibition), respectively. The ulcer area in 60 mg/kg group did not show superior anti-ulcerogenic potential than 40 mg/kg group as concerning the ulcer area. Meanwhile, 5 mg/kg group hardly protect gastric mucosa against the damage of ethanol by observation of ulcer area. Therefore, 10, 20 and 40 mg/kg of β-PAE were used in this study.

**Preliminary experiment for the concentration of ethanol**

Human GES-1 cells were kindly provided by the First Affiliated Hospital of Guangzhou University of Chinese Medicine. They were cultured in high glucose DMEM supplemented with 10% (v/v) heat-inactivated FBS at 37 °C in humidified atmosphere of 95 % air and 5 % $CO_2$.

To study the cytotoxicity of ethanol, GES-1 cells were divided into control group

and ethanol (0.5, 1, 2.5 and 5 %) groups, and the cell viability was determined by MTT assay. The GES-1 cells grown in the medium without ethanol were used as control.

As shown in Suppl. Figure S4, the tested concentration significantly reduced cell viability compared to the control group. And the $IC_{50}$ of ethanol was estimated to be around 1%. Thus 1% ethanol was chosen as the modeling concentration.

**The pH of gastric juice in rats with ethanol-induced gastric injury**

Male Sprague Dawley rats were divided into two groups of six animals each, and allowed to acclimatize to the conditions of laboratory for 7 days in the pre-experimental period. All rats were fasted, with access only to clean water for 24 h prior to experimentation. During experimentation, rats were administered with absolute ethanol by oral probe (0.5 mL/100g) to induce acute gastric injury. In contrast, the control group only received water. After one hour, all rats were euthanized and their stomachs were rapidly removed with ligation and opened. The pH was measured using pH meter.

As shown in Suppl. Figure S5A, the pH of gastric juice was 1.70 ± 0.32 in control group. By contrast, the pH of gastric juice was significantly increased in model group ($P<0.01$). It is indicated that damage in ethanol-induced gastric injury may cause the lack of gastric acid.

**Gastric metabolism of patchouli alcohol in simulated gastric juice at different pH**

The PA sample was prepared as described in the manuscript. Fifteen mL of

simulated gastric juice at pH 1.5 was poured into a conical flask with a stopper. And an identical conical flask was prepared as a negative control. Gastric metabolism of PA in simulated gastric juice at pH 2.5, 3.5, 4.5, 5.5 and 6.5 also perform the same operation. Twelve conical flasks were incubated on a shaker at 90 rpm in a humidified atmosphere (37 °C, 95 % air and 5 % $CO_2$) for 1 h. Then, 3 mL of PA sample was added to each conical flask simultaneously except the negative control group. After 60 min incubation, all conical flasks were removed. Subsequently, the mixture in a conical flask was extracted with 3 mL n-hexane, and the organic layer was dried with anhydrous sodium sulphate. Finally, one mL of organic extracts was subjected to GC-MS analysis. The experiment was repeated three times.

As shown in Suppl. Figure S5B and S5C, PA had been completely transformed in simulated gastric juice at pH 1.5 and 90.03% β-PAE was formed. In simulated gastric juice at pH 2.5, 7.42% of PA was converted into β-PAE. However, when the pH of simulated gastric juice was higher than 3.5, PA was not transformed at all.

Supporting References:


1 Zheng, Y. F. *et al.* Gastroprotective effect and mechanism of patchouli alcohol against ethanol, indomethacin and stress-induced ulcer in rats. *Chemico-Biological Interactions* **222**, 27-36 (2014).

2 Zhang, Z. B. *et al.* Anti-inflammatory activity of beta-patchoulene isolated from patchouli oil in mice. *European Journal of Pharmacology* **781**, 229-238 (2016).

**Figure Legends**

**Suppl. Figure S1. Gastric metabolism of patchouli alcohol *in vivo*.** (A) Total ion chromatogram (TIC) of PA and β-PAE after transformation of PA *in vivo* for 0, 30, 60, 90 and 120 min. (B) Time course of transformation of PA to β-PAE *in vivo*. Each point represents the means ± SD (*n*=6).

**Suppl. Figure S2. Diagram showing the design and time-course of experimental procedures for the preliminary experiment of administration time selection.**

**Suppl. Figure S3. Preliminary experiment for the administration time and dose selection.** Preliminary experiment for the administration time: (A) Macroscopic appearance of the gastric mucosa in ten groups (*n* = 6): Intact group (a), Vehicle+Ethanol (b), PA (20 mg/kg for 3 days)+Ethanol (c), PA (20 mg/kg for 5 days)+Ethanol (d), PA (20 mg/kg for 7 days)+Ethanol (e), PA (20 mg/kg for 9 days)+Ethanol (f), β-PAE (20 mg/kg for 3 days)+Ethanol (g), β-PAE (20 mg/kg for 5 days)+Ethanol (h), β-PAE (20 mg/kg for 7 days)+Ethanol (i), β-PAE (20 mg/kg for 9 days)+Ethanol (j). (B) Gastric ulcer areas of the gastric mucosa. (C) Ulcer inhibition (%). Preliminary experiment for the dose selection: (D) Macroscopic appearance of the gastric mucosa in seven groups (*n* = 6): Intact group (a), Vehicle+Ethanol (b), β-PAE (5 mg/kg)+Ethanol (c), β-PAE (10 mg/kg)+Ethanol (d), β-PAE (20 mg/kg)+Ethanol (e), β-PAE (40 mg/kg)+Ethanol (f), β-PAE (60 mg/kg)+Ethanol (g). (E) Gastric ulcer areas of the gastric mucosa. (F) Ulcer inhibition (%). The data are expressed as the mean ± SD and were analysed by ANOVA followed by LSD test.

**$P < 0.01$, *$P < 0.05$ versus the vehicle group. Student's t-test was performed to compare the intact and vehicle groups, ##$P < 0.01$.

**Suppl. Figure S4. Cytotoxicity of ethanol on cell viability of GES-1.** The data are expressed as the mean ± SD and were analysed by ANOVA followed by LSD test. **$P < 0.01$, *$P < 0.05$ versus the control group. (EtOH: ethanol)

**Suppl. Figure S5. Gastric metabolism of patchouli alcohol in simulated gastric juice at different pH.** (A) The pH of gastric juice in rats with ethanol-induced gastric injury ($n$=6). Student's t-test was performed to compare the control and model group, ##$P < 0.01$. (B) Time course of transformation of PA to β-PAE in simulated gastric juice at pH 1.5, 2.5, 3.5, 4.5, 5.5 and 6.5. Each point represents the means ± SD of three experiments. (C) Total ion chromatogram (TIC) of PA and β-PAE after transformation of PA in simulated gastric juice at pH 1.5, 2.5, 3.5, 4.5, 5.5 and 6.5 for 60 min.

**Suppl. Figure S6. Full-length blots/gels for Figure 6 are presented and cropping lines are indicated in red color.**

**Suppl. Figure S1.**

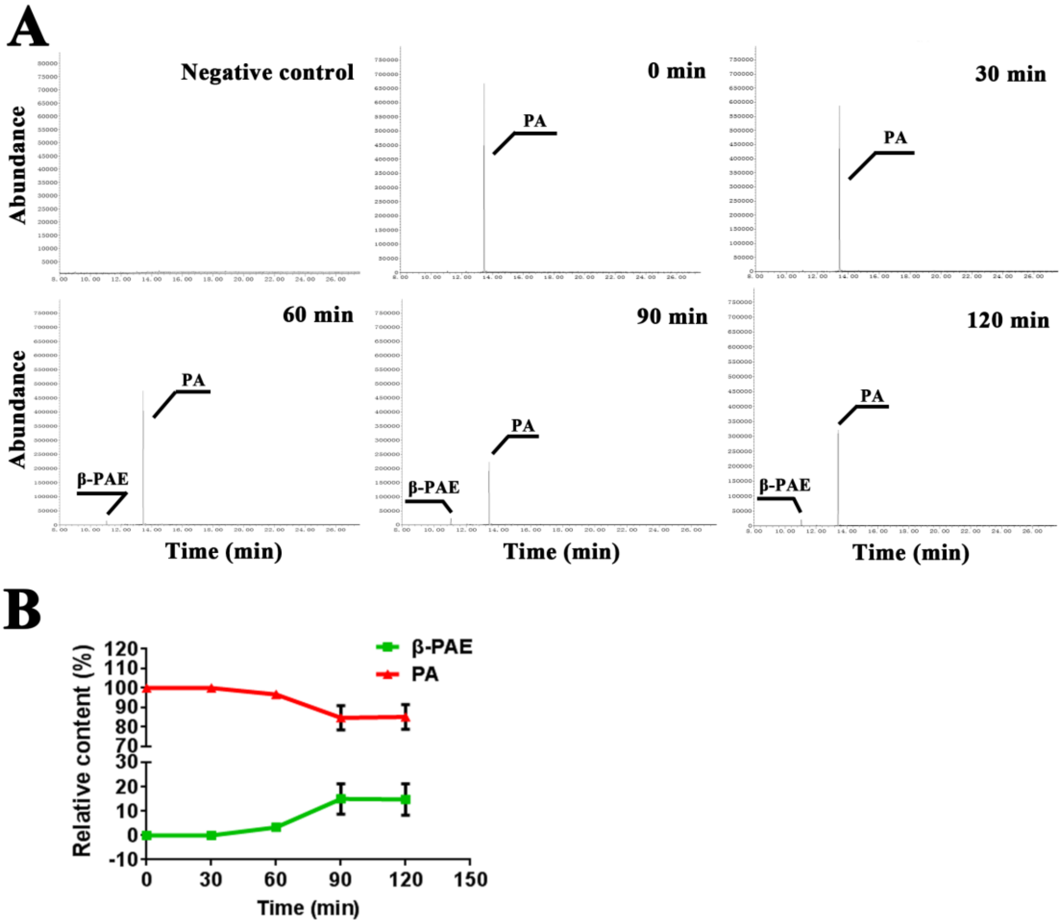

**Suppl. Figure S2.**

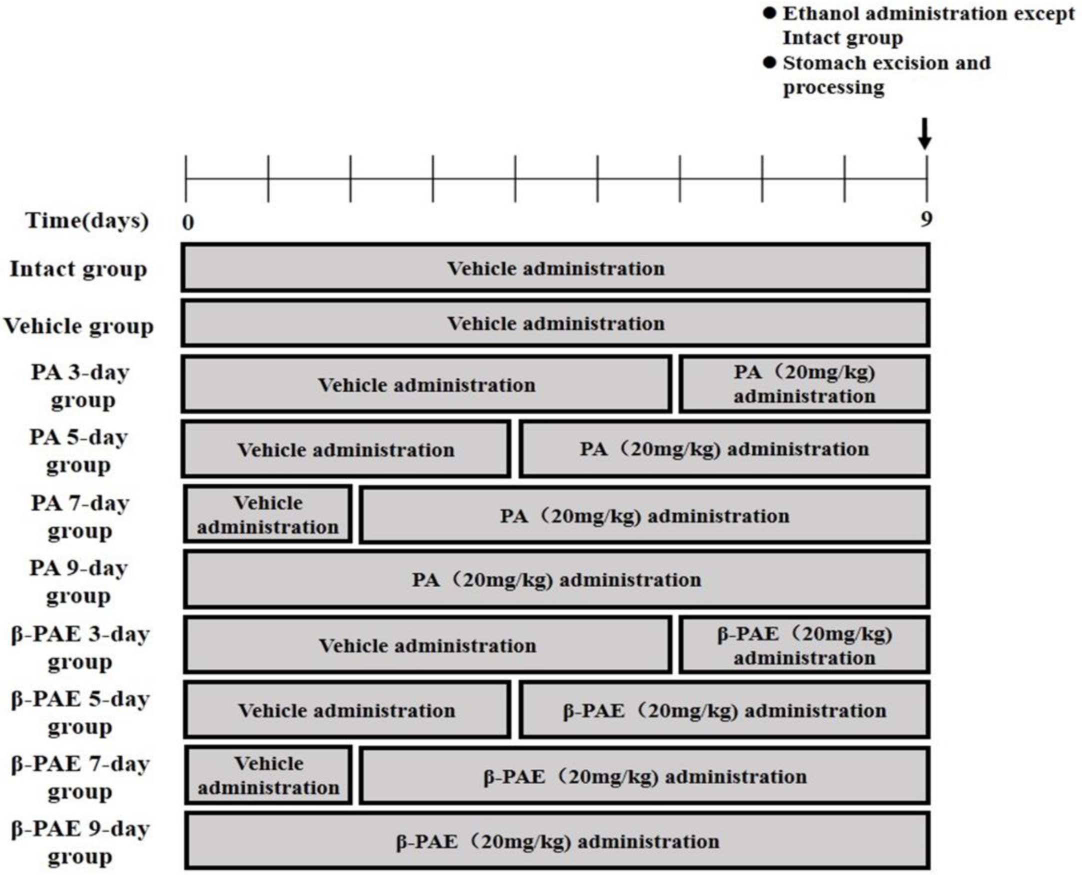

**Suppl. Figure S3.**

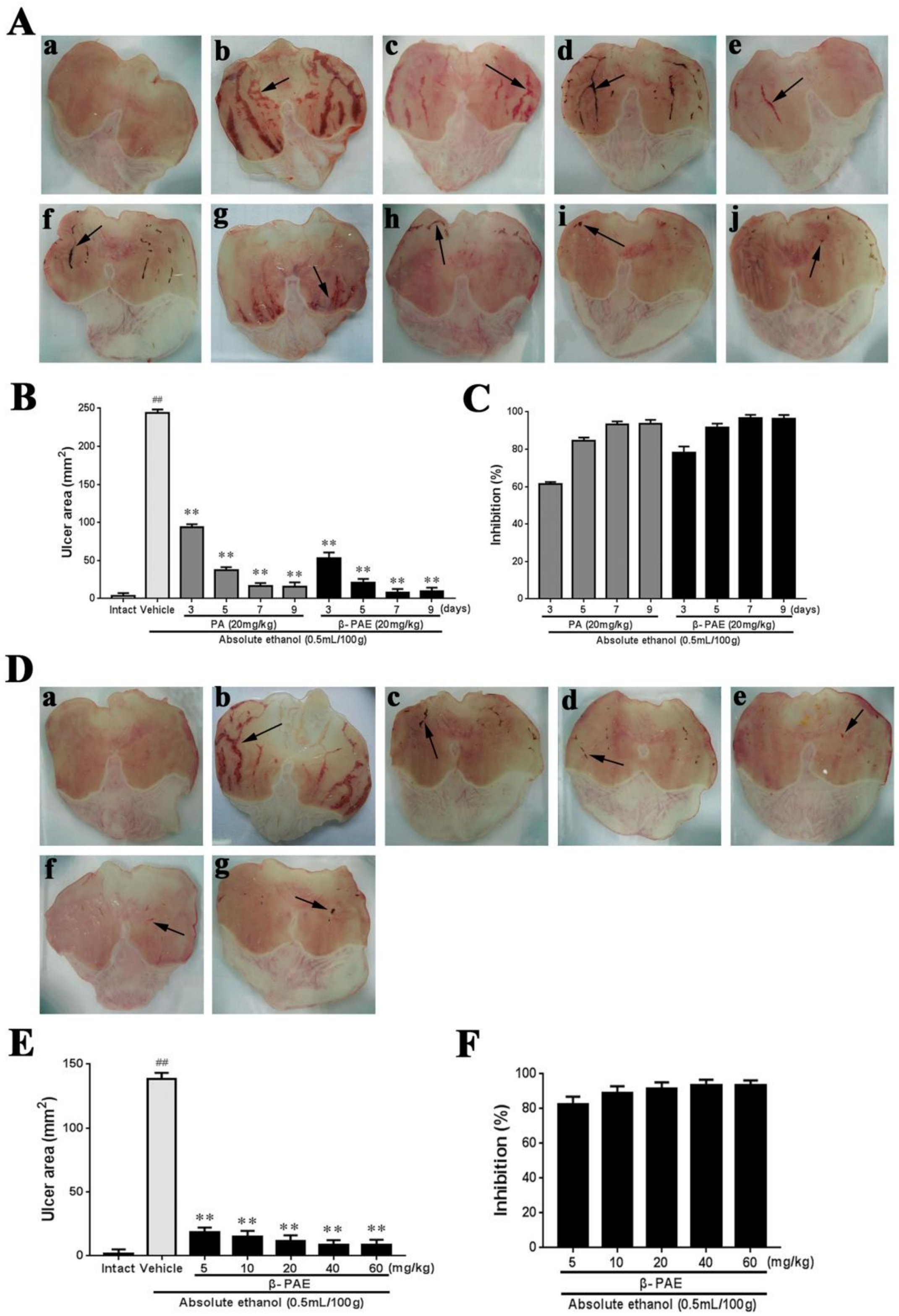

**Suppl. Figure S4.**

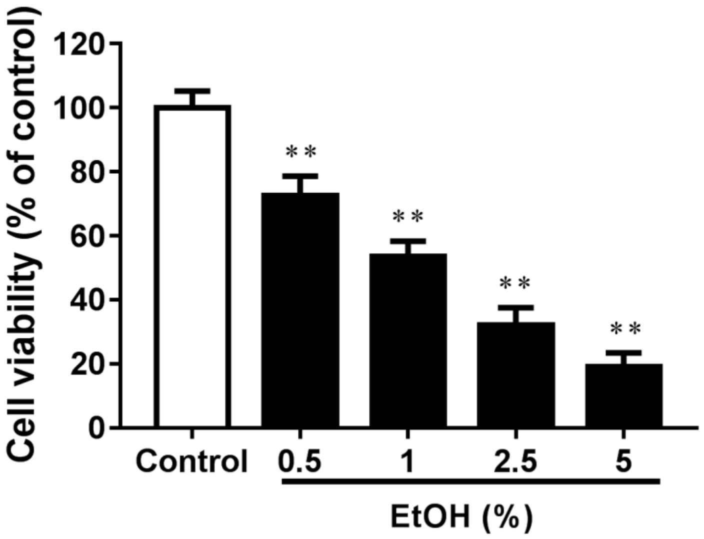

**Suppl. Figure S5.**

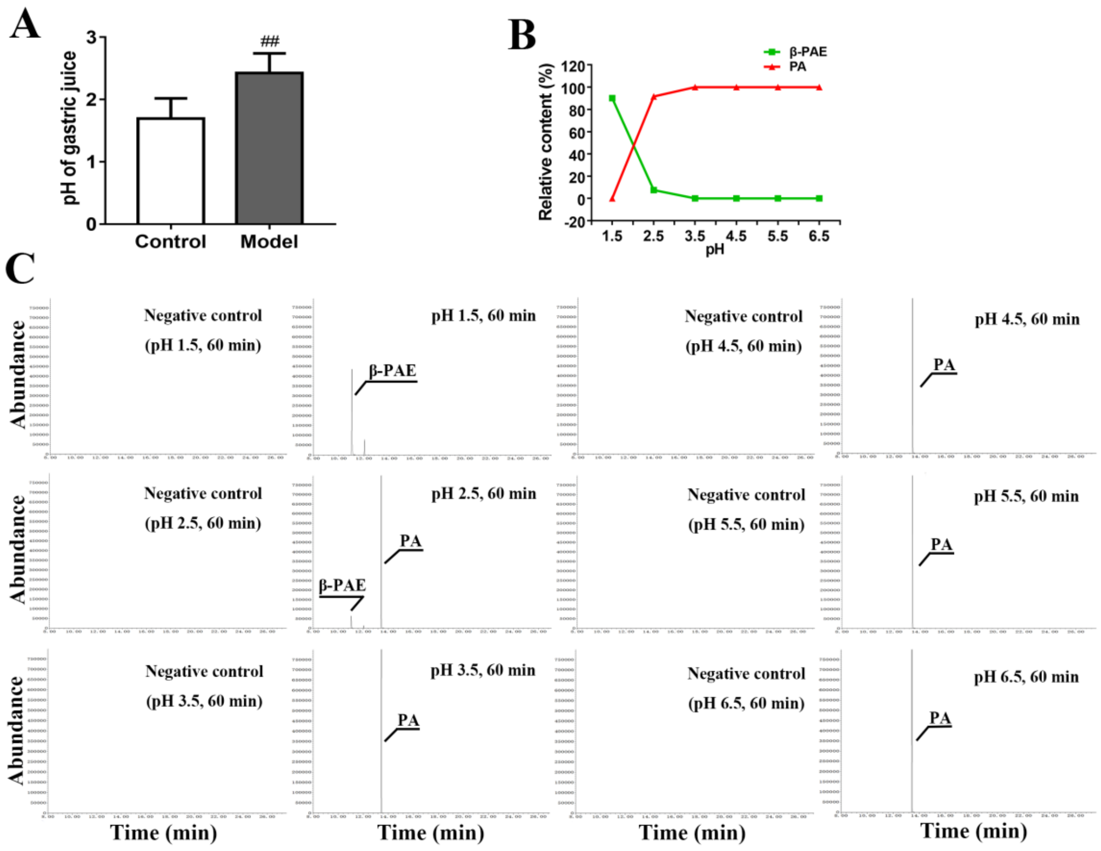

**Suppl. Figure S6.**

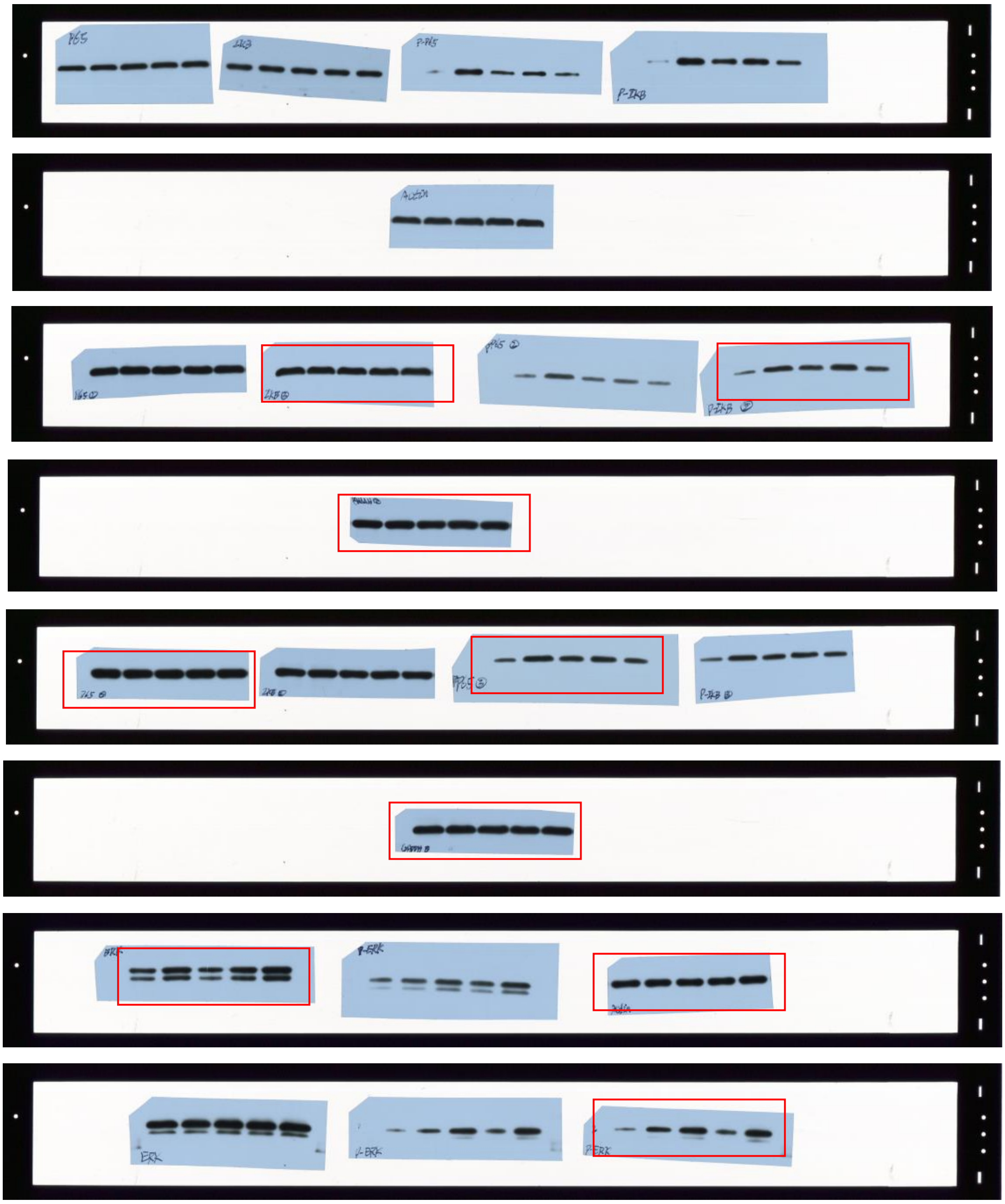

Supplement: Supplementary file 1 — Supplementary information [file 41598_2017_5996_MOESM1_ESM.pdf]
